# Validation of Self-reported Medical Condition in the Taiwan Biobank

**DOI:** 10.2188/jea.JE20240110

**Published:** 2025-03-05

**Authors:** Chi-Shin Wu, Le-Yin Hsu, Chen-Yang Shen, Wei J. Chen, Shi-Heng Wang

**Affiliations:** 1 National Center for Geriatrics and Welfare Research, National Health Research Institutes, Zhunan, Taiwan; 2 Department of Psychiatry, National Taiwan University Hospital, Yunlin Branch, Douliu, Taiwan; 3 Institute of Epidemiology and Preventive Medicine, College of Public Health, National Taiwan University, Taipei, Taiwan; 4 Graduate Program of Data Science, National Taiwan University and Academia Sinica, Taipei, Taiwan; 5 Institute of Biomedical Sciences, Academia Sinica, Taipei, Taiwan; 6 College of Public Health, China Medical University, Taichung, Taiwan; 7 Center for Neuropsychiatric Research, National Health Research Institutes, Miaoli, Taiwan; 8 Department of Medical Research, China Medical University Hospital, China Medical University, Taichung, Taiwan

**Keywords:** biobank, self-reported medical condition, concordance, claims data

## Abstract

**Background:**

This study aimed to validate self-reported medical conditions in the Taiwan Biobank (TWBB), in which participants were inquired about 30 disease conditions, by comparing them with claims records from Taiwan’s National Health Insurance (NHI) claims database.

**Methods:**

We identified 30 clinical diagnoses using International Classification of Diseases - Clinical Modification codes from ambulatory and hospital claims within the NHI claims database, matching diseases included in the TWBB. The concordance between self-reports and claims records was evaluated using tetrachoric correlation to assess the correlation between binary variables.

**Results:**

A total of 131,834 participants aged 30–70 years with data from the TWBB and NHI records were included. Concordance analysis revealed tetrachoric correlations ranged from 0.420 (chronic obstructive pulmonary disease) to 0.970 (multiple sclerosis). However, several disorders exhibited lower tetrachoric correlations. The concordance was higher among those with higher education attainment, and lower among married individuals.

**Conclusion:**

The concordance between self-reports in the TWBB and NHI claims records varied across clinical diagnoses, showing inconsistencies depending on participant characteristics. These findings underscore the need for further investigation, especially when these variables are crucial to research objectives. Integrating complementary databases, such as clinical diagnoses, prescription records, and medical procedures, can enhance accuracy through customized algorithms based on disease categories and participant characteristics and optimize sensitivity or positive predictive values to align with specific research objectives.

## INTRODUCTION

In the Big Data era, collecting extensive health information is essential for advancing biomedical research and precision medicine. Established in 2012, the Taiwan Biobank (TWBB) aims to improve Taiwanese health and promote biomedical research by enrolling 200,000 cancer-free individuals.^1^ The TWBB collects genetic data, self-reported medical conditions, lifestyle behaviors, and environmental risk factors.^2^ Self-reported conditions are crucial for understanding health status and predicting mortality and chronic disease. However, their accuracy may be reduced by cognitive ability, disease knowledge, and intentional withholding, leading to potential inaccuracies.^3^

Fortunately, the precision of self-reported data in the TWBB can be validated by cross-referencing it with Taiwan’s National Health Insurance (NHI) claims database. Taiwan’s comprehensive and mandatory NHI program, initiated in 1997, covers 99% of its 23.5 million residents. The NHI claims database spans 25 years and includes detailed demographic, diagnostic, procedural, and prescription information.^4^ The accuracy of clinical diagnoses in the NHI database is confirmed for common conditions, such as stroke, hypertension, diabetes, and major psychiatric disorders, demonstrating high sensitivity and positive predictive values.^4^^,^^5^

To assess the accuracy of self-reported medical conditions within the TWBB for future research, this study aimed to validate these self-reports by comparing them with the claims records in Taiwan’s NHI claims database encompassing disease conditions.

## METHODS

### Study samples

Since its inception in 2012, the TWBB has actively recruited study participants through advertising rather than random selection. The recruitment process spanned 30 distinct sites, encompassing community- and hospital-based locations strategically distributed in alignment with the population densities of various counties and cities across Taiwan. Data collection was conducted through face-to-face interviews with the participants. The data collection includes genetic data, acquiring demographic information, periodic assessments of self-reported medical conditions, performing physical examinations, blood and urine examinations, and assessment of socioeconomic status, lifestyle behaviors, and environmental factors. These assessments are conducted at regular intervals, usually every 2 to 4 years. As of the latest data available, 132,719 participants had been recruited from the TWBB study. Those lacking information on age, education level, or marital status were excluded. Ultimately, 131,834 participants were included in the study.

To be eligible for participation in the study, individuals were between 30 and 70 years old and had no history of cancer, based on the patient’s self-report and confirmed by the interviewer. Furthermore, each participant was required to provide informed consent, including an explicit agreement to link their data with external databases such as Taiwan’s NHI claims database. The Internal Review Board of the Taiwan Biobank approved the recruitment and sample collection procedures. This study was approved by the Central Regional Research Ethics Committee of the China Medical University, Taichung, Taiwan (approval number: CRREC-108-30).

### Self-reported medical conditions in TWBB

TWBB participants were asked, “Have you been diagnosed with any of the following disorders by a medical professional?” If the response was “yes,” the individual was categorized as having a disease. Information sourced from the TWBB assessed self-reported medical conditions, encompassing 30 conditions. These include cardiometabolic disorders (valvular heart disease, coronary artery disease, arrhythmia, cardiomyopathy, congenital heart disease, hyperlipidemia, hypertension, stroke, and type II diabetes mellitus), respiratory disorders (asthma and chronic obstructive pulmonary disease [COPD]), gastrointestinal disorders (peptic ulcer disease, gastroesophageal reflux disease, and irritable bowel syndrome), psychiatric disorders (schizophrenia, bipolar disorder, major depressive disorder, substance use disorder, and obsessive-compulsive disorder), neurological disorders (epilepsy, migraine, multiple sclerosis, Parkinson’s disease, dementia), orthopedic disorders (osteoporosis, rheumatoid arthritis, osteoarthritis, and gout), and urinary disorders (chronic kidney disease and renal stone).

### Claims-recorded measures

The NHI claims database incorporates International Classification of Diseases (ICD) diagnostic codes, transitioning from ICD-9 to ICD-10 in 2016. We used ICD-Clinical Modification (CM) codes to identify 30 clinical diagnoses from ambulatory and hospital claims within the NHIRD. The ICD codes for the clinical diagnoses featured in the TWBB are shown in eTable 1. We adopted a more robust method to address the potential inaccuracies of using a single claims record for clinical diagnosis, which may result in provisional or incorrect identifications. Our approach uses a commonly employed algorithm that requires at least two outpatient claims or one discharge claim within 2 years the observational period before the initial interview date to confirm a diagnosis.^4^ Although other strategies—like verifying related medications, restricting diagnoses to specialist consultations, or linking to additional databases—can enhance diagnostic reliability, they are not universally applicable.

### Statistical analysis

We employed tetrachoric correlation as the primary analysis to assess the concordance between self-reports and claims records. The interpretation of the tetrachoric correlation was ≤0.25 as slight, 0.26–0.50 as fair, 0.51–0.75 as moderate, and >0.75 as good to excellent.^6^ Using claims records as the ‘gold’ standard, we reported the sensitivity, specificity, and positive and negative predictive values for self-reported health conditions in a secondary analysis. We conducted several sensitivity analyses, including Cohen’s kappa statistic, 5-year observational period for claims records, and the influence of transitioning ICD version since 2016, and showed the details in eMaterial 1. SAS version 9.4 (SAS Institute, Cary, NC, USA) was utilized for all statistical analyses.

## RESULTS

Baseline characteristics are summarized in Table 1, with roughly equal distribution across age groups. Approximately 63.8% were female, 73.0% married, and 87.6% had ≥12 years of education.

**Table 1.** Baseline characteristics of study participants in Taiwan Biobank (*n* = 131,834)

Characteristics	*n* (%)
Gender	
Female	84,101 (63.79)
Male	47,733 (36.21)
Age group, years	
30–39	30,370 (23.04)
40–49	31,963 (24.24)
50–59	38,952 (29.55)
60–70	30,549 (23.17)
Education	
Illiterate/self-educated	286 (0.22)
Elementary school	6,429 (4.88)
Junior high school	9,607 (7.29)
Senior high school/Vocational high school	38,283 (29.04)
University/College	62,614 (47.49)
Graduate school or above	14,615 (11.09)
Marriage	
Single	18,531 (14.06)
Married	96,294 (73.04)
Divorced/Separated	11,144 (8.45)
Widowed	5,865 (4.45)

Table 2 shows that the top self-reported conditions were peptic ulcer disease, gastroesophageal reflux disease, and hypertension; the top claims-recorded diagnoses were hyperlipidemia, hypertension, and osteoarthritis. Tetrachoric correlation coefficients ranged from 0.42 to 0.97, indicating good-to-excellent concordance for various diseases, including coronary heart disease, hypertension, stroke, type II diabetes mellitus, schizophrenia, major depressive disorder, obsessive-compulsive disorders, epilepsy, multiple sclerosis, Parkinson’s disease, chronic kidney disease, rheumatoid arthritis, and gout. However, COPD and irritable bowel syndrome demonstrated only fair concordance. Given the prevalence of most diseases was relatively low, the specificity and negative predictive value were almost all >0.90. The sensitivity generally was low, ranging from 0.02 for substance use disorder to 0.69 for hypertension. The positive predictive value ranged from 0.03 for cardiomyopathy to 0.89 for hypertension.

**Table 2.** Tetrachoric correlation, sensitivity, specificity, positive predictive value, negative predictive value for self-reports and claims records of chronic diseases among study participants in Taiwan Biobank

	Self-reported Prevalence	Claims-records prevalence	Tetrachoric correlation	Sensitivity	Specificity	Positive predictive value	Negative predictive value
**Cardiometabolic disorder**							
Valvular heart disease	4.22	1.57	0.585	0.38	0.96	0.14	0.99
Coronary artery disease	1.28	4.38	**0.820**	0.22	1.00	0.74	0.97
Arrhythmia	4.48	2.57	0.638	0.39	0.96	0.23	0.98
Cardiomyopathy	0.77	0.05	0.614	0.40	0.99	0.03	1.00
Congenital heart disease	0.20	0.08	0.585	0.13	1.00	0.05	1.00
Hyperlipidemia	7.49	16.4	0.721	0.32	0.97	0.69	0.88
Hypertension	12.18	15.78	**0.950**	0.69	0.98	0.89	0.94
Stroke	0.63	1.74	**0.820**	0.22	1.00	0.62	0.99
Type II Diabetes Mellitus	5.00	7.81	**0.971**	0.60	1.00	0.94	0.97
**Respiratory disorder**							
Asthma	3.59	2.83	0.693	0.39	0.97	0.30	0.98
Chronic obstructive pulmonary disease	1.12	1.84	0.420	0.09	0.99	0.14	0.98
**Gastrointestinal disorder**							
Peptic ulcer disease	14.47	9.71	0.523	0.44	0.89	0.29	0.94
Gastroesophageal reflux disease	13.87	8.63	0.634	0.53	0.90	0.33	0.95
Irritable bowel syndrome	2.51	2.07	0.487	0.19	0.98	0.16	0.98
**Psychiatric disorder**							
Schizophrenia	0.20	0.31	**0.953**	0.48	1.00	0.75	1.00
Bipolar disorder	0.68	0.49	0.748	0.33	0.99	0.24	1.00
Major depressive disorder	3.60	3.54	**0.758**	0.42	0.98	0.41	0.98
Substance use disorder	0.04	0.91	0.639	0.02	1.00	0.50	0.99
Obsessive-compulsive disorder	0.11	0.11	**0.825**	0.27	1.00	0.30	1.00
**Neurological disorder**							
Epilepsy	0.35	0.28	**0.894**	0.54	1.00	0.43	1.00
Migraine	2.89	1.08	0.631	0.39	0.97	0.14	0.99
Multiple sclerosis	0.02	0.02	**0.970**	0.50	1.00	0.50	1.00
Parkinson’s disease	0.11	0.11	**0.943**	0.55	1.00	0.55	1.00
Dementia	0.03	0.16	0.737	0.06	1.00	0.33	1.00
**Orthopedic disorder**							
Osteoporosis	3.96	1.75	0.648	0.42	0.97	0.19	0.99
Rheumatoid arthritis	0.71	0.79	**0.834**	0.39	1.00	0.43	1.00
Osteoarthritis	3.83	9.98	0.614	0.20	0.98	0.52	0.92
Gout	3.86	3.22	**0.815**	0.53	0.98	0.44	0.98
**Urinary disorder**							
Chronic kidney disease	0.13	0.9	**0.832**	0.11	1.00	0.77	0.99
Renal stone	6.34	2.54	0.732	0.59	0.95	0.24	0.99

Bold font indicates good concordance when tetrachoric correlation >0.75.

Table 3 details the sex-, age-, education-, and marital status-stratified concordance. Tetrachoric correlations for conditions like hyperlipidemia, hypertension, stroke, peptic ulcer disease, gastroesophageal reflux disease, bipolar disorder, migraine, gout, and renal stone were higher in individuals under 50 years but lower for arrhythmia and osteoarthritis compared to those 50 years and older. Tetrachoric correlations for certain disorders were higher among men, while for others, it was higher among women, with some showing no difference. Participants with at least 12 years of education exhibited higher concordance for several disorders, including coronary artery disease and chronic kidney disease, compared to those with less education. Single, divorced, or widowed individuals showed higher concordance for bipolar disorder, osteoporosis, osteoarthritis, and gout compared to those partnered.

**Table 3.** Tetrachoric correlation between self-reports and claims records of chronic diseases, stratified by gender, age, education, and marital status

Tetrachoric correlation	Gender		Age, years		Educations, years		Marital Status	
	Female	Male	<50	≥50	≤12	>12	Single/divorced/ widowed	Married
**Cardiometabolic disorder**								
Valvular heart disease	0.588 (0.019)	0.576 (0.019)	0.613 (0.017)	0.590 (0.012)	0.601 (0.015)	0.583 (0.013)	0.586 (0.019)	0.586 (0.012)
Coronary artery disease	0.725 (0.013)	**0.864 (0.007)**	0.796 (0.019)	0.800 (0.007)	0.792 (0.008)	**0.845 (0.008)**	0.813 (0.013)	0.821 (0.007)
Arrhythmia	0.599 (0.010)	**0.701 (0.011)**	0.575 (0.017)	**0.644 (0.009)**	0.624 (0.011)	0.649 (0.011)	0.615 (0.016)	0.646 (0.009)
Cardiomyopathy	0.681 (0.049)	0.547 (0.055)	0.481 (0.114)	0.621 (0.041)	0.546 (0.057)	0.679 (0.048)	0.611 (0.084)	0.614 (0.041)
Congenital heart disease	0.589 (0.058)	0.587 (0.072)	0.655 (0.062)	0.540 (0.066)	0.552 (0.077)	0.606 (0.056)	0.595 (0.052)	0.585 (0.053)
Hyperlipidemia	**0.741 (0.005)**	0.692 (0.007)	**0.740 (0.008)**	0.666 (0.009)	0.677 (0.006)	**0.754 (0.005)**	0.724 (0.008)	0.719 (0.005)
Hypertension	**0.957 (0.001)**	0.939 (0.002)	**0.951 (0.002)**	0.938 (0.002)	0.944 (0.002)	**0.954 (0.002)**	0.951 (0.002)	0.950 (0.001)
Stroke	0.755 (0.011)	**0.861 (0.010)**	**0.870 (0.017)**	0.795 (0.010)	0.811 (0.012)	0.827 (0.012)	0.823 (0.016)	0.819 (0.010)
Type II Diabetes Mellitus	0.970 (0.002)	0.971 (0.002)	0.971 (0.003)	0.966 (0.001)	0.968 (0.002)	0.972 (0.002)	0.973 (0.002)	0.970 (0.001)
**Respiratory Disorder**								
Asthma	**0.709 (0.008)**	0.662 (0.013)	0.693 (0.011)	0.711 (0.009)	0.706 (0.010)	0.696 (0.009)	0.683 (0.014)	0.699 (0.008)
Chronic obstructive pulmonary disease	0.356 (0.030)	**0.474 (0.022)**	0.367 (0.036)	0.419 (0.019)	0.435 (0.024)	0.416 (0.025)	0.416 (0.033)	0.423 (0.019)
**Gastrointestinal disorder**								
Peptic ulcer disease	0.526 (0.007)	0.524 (0.009)	**0.562 (0.009)**	0.479 (0.007)	0.508 (0.008)	**0.531 (0.007)**	0.537 (0.010)	0.518 (0.006)
Gastroesophageal reflux disease	0.626 (0.006)	**0.650 (0.008)**	**0.642 (0.008)**	0.622 (0.006)	0.622 (0.007)	**0.644 (0.006)**	0.645 (0.009)	0.631 (0.005)
Irritable bowel syndrome	0.442 (0.016)	**0.547 (0.017)**	0.512 (0.015)	0.481 (0.016)	0.479 (0.019)	0.506 (0.015)	0.459 (0.024)	0.497 (0.013)
**Psychiatric disorder**								
Schizophrenia	0.946 (0.009)	0.962 (0.008)	0.959 (0.007)	0.936 (0.013)	0.942 (0.010)	0.963 (0.010)	0.956 (0.007)	0.922 (0.017)
Bipolar disorder	0.748 (0.017)	0.746 (0.024)	**0.790 (0.017)**	0.703 (0.021)	0.668 (0.025)	**0.803 (0.015)**	**0.784 (0.018)**	0.703 (0.021)
Major depressive disorder	0.752 (0.007)	0.765 (0.011)	0.764 (0.009)	0.752 (0.007)	0.759 (0.008)	0.754 (0.008)	0.757 (0.009)	0.753 (0.007)
Substance use disorder	0.556 (0.115)	0.612 (0.045)	0.673 (0.043)	0.524 (0.090)	0.671 (0.043)	0.533 (0.090)	0.666 (0.050)	0.600 (0.062)
Obsessive-compulsive disorder	0.830 (0.030)	0.818 (0.036)	0.856 (0.025)	0.746 (0.051)	0.843 (0.036)	0.815 (0.030)	0.814 (0.035)	0.830 (0.031)
**Neurological disorder**								
Epilepsy	0.880 (0.015)	0.905 (0.013)	0.900 (0.013)	0.889 (0.015)	0.895 (0.014)	0.895 (0.013)	0.910 (0.014)	0.881 (0.014)
Migraine	0.616 (0.025)	0.649 (0.024)	**0.673 (0.014)**	0.589 (0.016)	0.593 (0.017)	**0.662 (0.014)**	0.643 (0.020)	0.626 (0.020)
Multiple sclerosis	0.968 (0.017)	0.977 (0.018)	0.993 (0.006)	0.909 (0.046)	0.915 (0.056)	0.984 (0.010)	0.972 (0.018)	0.968 (0.018)
Parkinson’s disease	0.939 (0.015)	0.947 (0.013)	0.955 (0.032)	0.943 (0.011)	0.934 (0.015)	0.952 (0.013)	0.896 (0.032)	0.955 (0.010)
Dementia	0.681 (0.067)	0.797 (0.055)	NA	0.751 (0.045)	0.715 (0.072)	0.763 (0.068)	0.691 (0.084)	0.761 (0.050)
**Orthopedic disorder**								
Osteoporosis	**0.643 (0.010)**	0.588 (0.025)	0.532 (0.042)	0.601 (0.010)	0.594 (0.012)	**0.699 (0.012)**	**0.675 (0.015)**	0.637 (0.010)
Rheumatoid arthritis	**0.851 (0.009)**	0.734 (0.029)	0.833 (0.018)	0.828 (0.011)	0.839 (0.012)	0.826 (0.013)	0.803 (0.020)	0.844 (0.010)
Osteoarthritis	0.612 (0.007)	0.603 (0.013)	0.506 (0.022)	**0.558 (0.008)**	0.588 (0.009)	**0.621 (0.009)**	**0.637 (0.012)**	0.606 (0.007)
Gout	0.618 (0.021)	**0.799 (0.006)**	**0.851 (0.007)**	0.791 (0.007)	0.786 (0.008)	**0.836 (0.006)**	0.799 (0.011)	0.818 (0.005)
**Urinary disorder**								
Chronic kidney disease	0.837 (0.022)	0.821 (0.021)	0.854 (0.026)	0.826 (0.018)	0.845 (0.022)	0.826 (0.020)	**0.876 (0.022)**	0.811 (0.020)
Renal stone	0.736 (0.000)	0.704 (0.009)	**0.770 (0.009)**	0.703 (0.008)	0.729 (0.009)	0.734 (0.009)	0.741 (0.013)	0.728 (0.007)

Values reported as mean (standard error). Bold font indicates that the correlation is statistically significantly higher than that of the counterpart group using the z-test (*P*-value <0.05).

The results of sensitivity analyses present in eTable 2, eTable 3, and eMaterial 1 suggested the robustness of our findings.

## DISCUSSION

This study found varying levels of concordance between self-reports from TWBB and NHI claims records across different clinical diagnoses influenced by study subject characteristics. Concordance was good to excellent for common cardiometabolic diseases, like hypertension and diabetes mellitus, which is consistent with previous surveys.^7^ However, concordance for COPD and irritable bowel syndrome was only fair. The low concordance for COPD may be due to its vague and poorly understood nature among the Taiwanese population, leading to low self-reported prevalence. For irritable bowel syndrome, common symptoms often overlap with other gastrointestinal disorders and are frequently attributed to irritable bowel syndrome, resulting in a higher self-reported prevalence compared to claims records. This discrepancy likely explains the lower correlation observed.

For psychiatric disorders, schizophrenia, major depressive disorders, and obsessive-compulsive disorders, they exhibited good-to-excellent concordance, likely due to the severity and chronic nature of these diagnoses, which make them less susceptible to misreporting. In contrast, substance use disorders displayed significantly lower concordance, likely due to underreporting.^8^

In neurological disorders, high concordance was noted for multiple sclerosis, epilepsy, and Parkinson’s disease, while migraine agreement was moderate. Some patients experiencing various types of headaches may misidentify their condition as a migraine when self-diagnosing, while individuals with dementia often lack insight into their condition.^9^

Orthopedic disorders generally exhibited good-to-excellent concordance, with rheumatoid arthritis and gout having reasonable agreement. Osteoporosis was underreported in self-reports compared to claims records, possibly due to screening practices and NHI prescription restrictions; anti-osteoporosis medications were reimbursed only for severe osteoporosis with hip or vertebral fractures.^10^ Conversely, osteoarthritis was more prevalent in claims records, possibly due to underreporting of mild symptoms.

Substance use disorders, dementia, and COPD showed low sensitivity, suggesting that self-reporting poorly captured these diseases, which could lead to an underestimation of their prevalence. Additionally, the low positive predictive value may arise from misconceptions about the diseases or from individuals having recovered or not receiving ongoing treatment. However, the high specificity and negative predictive value of self-reports make them reliable for ruling out conditions in research settings, supporting their use in studies requiring confirmation of non-disease.

### Concordance of self-reported diagnosis varied with the characteristics of subjects

The tetrachoric correlations vary across different diseases. Due to word constraints, it was not feasible to address each disease individually. In general, higher concordance rates were observed in some, but not all, disorders among participants younger than 50 years, those with at least 12 years of education, and individuals who are single, divorced, or widowed. Older adults with more complex health conditions and memory impairment show lower concordance between self-reports and claims records.^7^^,^^11^ Educational attainment also influences concordance,^12^ with higher levels correlating with better agreement. Being single was associated with higher tetrachoric correlations, possibly due to family involvement in medical decisions in Taiwan.^13^

Self-reported information accuracy varied among participant characteristics, which is consistent with other studies.^7^^,^^14^^–^^16^ For example, assessing self-reported hypertension accuracy revealed significant variations in sensitivity and specificity across countries and age groups.^15^ Similarly, validating self-reported diabetes mellitus showed increased false positives among females, older individuals, smokers, and those with a family history of diabetes.^16^

### Validation of self-reported information in other biobank databases

Research on the accuracy of self-reported information within biobanks is limited. One study compared data from the United Kingdom Biobank with clinical records from Oxford’s clinical record search to assess its suitability for mental health research. Results showed varying positive predictive values for self-reported dementia, depression, bipolar disorder, and schizophrenia.^17^ Another study evaluated the accuracy of identifying refractive errors within the United Kingdom Biobank using self-reported data on the age at which the glasses or contact lenses were first used. Self-reported information on optical correction was found reliable, particularly for identifying cases of myopia.^18^ However, validation studies for self-reported information in biobanks have primarily focused on the United Kingdom Biobank. This study represents the first attempt to validate self-reported medical conditions within the TWBB, suggesting further investigation into the accuracy of self-reported information in this cohort is warranted.

### Limitations and strengths of the study

This study has several notable limitations. First, it lacks a definitive “gold standard” for clinical diagnoses. While cardiometabolic and psychiatric disorders in the NHI claims records are well-validated,^4^^,^^5^ the accuracy of diagnoses for neurological, gastrointestinal, and orthopedic disorders remains uncertain and unvalidated. Second, the TWBB relies on self-reported data, and NHI data depend on treatment-seeking behavior, which could increase the false negative rate due to under-reporting and under-treatment. Third, the TWBB sample was recruited through advertising, not random selection, raising concerns about the representativeness and generalizability of the findings. Further assessment of the sample’s demographic diversity and its applicability to the general population is needed. Finally, patients with existing cancer were excluded, so the concordance of cancer diagnoses was not evaluated, highlighting the need for future studies on the accuracy of self-reported cancer diagnoses in similar settings.

### Conclusion

The concordance between self-reports in the TWBB and the NHI claims records was moderate to excellent for most clinical diagnoses. However, inconsistencies in specific disease categories were noted and required further investigation. Future studies using TWBB data should be cautious with self-reported diseases with low concordance. Enhancing accuracy may be achieved by integrating complementary databases—such as clinical diagnoses, prescription records, and medical procedures from the NHI claims database—and developing specific algorithms to combine these sources. Additionally, concordance varied with participant characteristics, suggesting that customizing algorithms based on these characteristics could optimize sensitivity and positive predictive values to meet the study’s aims and objectives better.

## SUPPLEMENTARY MATERIAL

eMaterial 1. Supplemental methods, results, and discussion

eTable 1. The ICD codes for 30 common clinical diagnoses

eTable 2. Kappa statistics and tetrachoric correlation between self-reports and claims records of chronic diseases using 2- and 5-year observational periods

eTable 3. Tetrachoric correlation between self-reports and claims records of chronic diseases using ICD-9 and ICD-10
