# Supplementary material for: Validation of Self-reported Medical Condition in the Taiwan Biobank
Source: J Epidemiol. 2025 Mar 5;35(3):141–6. doi: 10.2188/jea.JE20240110 (PMC11821376; doi:10.2188/jea.JE20240110)
Supplement: Supplementary file 1 [file je-35-141-s001.pdf]

eTable 1. The ICD codes for 30 common clinical diagnoses

	ICD-9	ICD-10
**Cardiometabolic disorder**		
Valvular heart disease	394-397, 424	I05-I08, I34-I37
Coronary artery disease	410-414	I20-I25
Arrhythmia	427	I46.2, I46.8, I46.9, I47, I47.1, I47.2, I47.9, I48, I48.1, I48.2, I48.3, I48.4, I48.91, I48.92, I49.01, I49.02, I49.1, I49.2, I49.3, I49.4, I49.5, I49.8, I49.9
Cardiomyopathy	425	I42-I43
Congenital heart disease	746	Q22-Q24
Hyperlipidemia	272	E78
Hypertension	401-405	I10, I11, I12, I13, I15
Stroke	430-438	I60-I69
Type II Diabetes Mellitus	250 (exclude 250.x1, 250.x3)	E08, E09, E11, E13
**Respiratory disorder**		
Asthma	493	J45
Chronic obstructive pulmonary disease	491, 492, 496	J41-J44
**Gastrointestinal disorder**		
Peptic ulcer disease	531-534	K25-K28
Gastroesophageal reflux disease	530.11, 530.81	K21
Irritable bowel syndrome	564.1	K58
**Psychiatric disorder**		
Schizophrenia	295.x	F20, F25
Bipolar disorder	296.0-296.1, 296.4-296.8	F30, F31, F34.0,
Major depressive disorder	296.2x, 296.3x, 300.4, 311	F32, F33, F34.1
Substance use disorder	291, 292, 303.0, 303.9, 304, 305	F10-F19
Obsessive-compulsive disorder	300.3	F42
**Neurological disorder**		
Epilepsy	345	G40
Migraine	346	G43
Multiple sclerosis	340	G35
Parkinson's disease	332.0	G20
Dementia	290, 294.1, 331.0-331.2	F00-F03, G30, G31.1
**Orthopedic disorder**		
Osteoporosis	733.0, 733.1	M80.00XA, M80.80XA, M84.40XA, M84.50XA, M84.60XA, M80.021A, M80.022A, M80.029A, M80.821A, M80.822A, M80.829A, M84.421A, M84.422A, M84.429A, M84.521A, M84.522A, M84.529A, M84.621A, M84.622A, M84.629A, M80.031A, M80.032A, M80.039A, M80.831A, M80.832A, M80.839A, M84.431A, M84.432A, M84.433A, M84.434A, M84.439A, M84.531A, M84.532A, M84.533A, M84.534A, M84.539A, M84.631A, M84.632A, M84.633A, M84.634A, M84.639A, M48.50XA,

		M48.51XA, M48.52XA, M48.53XA, M48.54XA, M48.55XA, M48.56XA, M48.57XA, M48.58XA, M80.08XA, M80.88XA, M84.48XA, M84.58XA, M84.68XA, M80.051A, M80.052A, M80.059A, M80.851A, M80.852A, M80.859A, M84.451A, M84.452A, M84.459A, M84.551A, M84.552A, M84.553A, M84.559A, M84.651A, M84.652A, M84.653A, M84.659A, M80.051A, M80.052A, M80.059A, M80.851A, M80.852A, M80.859A, M84.451A, M84.452A, M84.453A, M84.551A, M84.552A, M84.553A, M84.559A, M84.651A, M84.652A, M84.653A, M84.659A, M80.061A, M80.062A, M80.069A, M80.071A, M80.072A, M80.079A, M80.861A, M80.862A, M80.869A, M80.871A, M80.872A, M80.879A, M84.361A, M84.362A, M84.363A, M84.364A, M84.369A, M84.461A, M84.462A, M84.463A, M84.464A, M84.469A, M84.471A, M84.472A, M84.473A, M84.561A, M84.562A, M84.563A, M84.564A, M84.569A, M84.571A, M84.572A, M84.573A, M84.661A, M84.662A, M84.663A, M84.664A, M84.669A, M84.671A, M84.672A, M84.673A, M80.011A, M80.012A, M80.019A, M80.041A, M80.042A, M80.049A, M80.071A, M80.072A, M80.079A, M80.811A, M80.812A, M80.819A, M80.841A, M80.842A, M80.849A, M80.871A, M80.872A, M80.879A, M84.311A, M84.312A, M84.319A, M84.321A, M84.322A, M84.331A, M84.332A, M84.333A, M84.334A, M84.341A, M84.342A, M84.343A, M84.344A, M84.345A, M84.346A, M84.350A, M84.351A, M84.352A, M84.371A, M84.372A, M84.374A, M84.375A, M84.376A, M84.377A, M84.378A, M84.379A, M84.38xA, M84.411A, M84.412A, M84.419A, M84.441A, M84.442A, M84.443A, M84.444A, M84.445A, M84.446A, M84.454A, M84.474A, M84.475A, M84.476A, M84.477A, M84.478A, M84.479A, M84.48XA, M84.511A, M84.512A, M84.519A, M84.541A, M84.542A, M84.549A, M84.550A, M84.574A, M84.575A, M84.576A, M84.611A, M84.612A, M84.619A, M84.641A, M84.642A, M84.649A, M84.650A, M84.674A, M84.675A, M84.676A, M84.68XA, M81
Rheumatoid arthritis	714.0	M05-M06
Osteoarthritis	715	M15-M19
Gout	274	M10
**Urinary disorder**		
Chronic kidney disease	585	N18
Renal stone	592, 594	N20-N22

ICD, International Classification of Diseases.

eTable 2. Kappa statistics and tetrachoric correlation between self-reports and claims records of chronic diseases using 2- and 5-year observational periods

		2-year observational periods			5-year observational periods		
	Self-reported Prevalence	Claims-records prevalence	Kappa	Tetrachoric correlation	Claims-records prevalence	Kappa	Tetrachoric correlation
**Cardiometabolic disorder**							
Valvular heart disease	4.22	1.57	0.186	0.585	2.89	0.270	0.635
Coronary artery disease	1.28	4.38	0.322	0.820	6.82	0.247	0.813
Arrhythmia	4.48	2.57	0.263	0.638	4.50	0.297	0.638
Cardiomyopathy	0.77	0.05	0.048	0.614	0.08	0.064	0.608
Congenital heart disease	0.20	0.08	0.081	0.585	0.13	0.094	0.580
Hyperlipidemia	7.49	16.40	0.370	0.721	22.13	0.329	0.734
Hypertension	12.18	15.78	0.739	0.950	18.47	0.704	0.955
Stroke	0.63	1.74	0.326	0.820	2.93	0.249	0.806
Type II Diabetes Mellitus	5.00	7.81	0.720	0.971	9.50	0.645	0.967
**Respiratory disorder**							
Asthma	3.59	2.83	0.319	0.693	5.02	0.335	0.694
Chronic obstructive pulmonary disease	1.12	1.84	0.095	0.420	3.70	0.088	0.414
**Gastrointestinal disorder**							
Peptic ulcer disease	14.47	9.71	0.266	0.523	17.75	0.327	0.569
Gastroesophageal reflux disease	13.87	8.63	0.338	0.634	14.68	0.405	0.669
Irritable bowel syndrome	2.51	2.07	0.151	0.487	4.46	0.171	0.501
**Psychiatric disorder**							
Schizophrenia	0.2	0.31	0.594	0.953	0.36	0.559	0.949
Bipolar disorder	0.68	0.49	0.273	0.748	0.73	0.289	0.749
Major depressive disorder	3.6	3.54	0.397	0.758	5.59	0.415	0.777
Substance use disorder	0.04	0.91	0.039	0.639	1.64	0.026	0.628
Obsessive-compulsive disorder	0.11	0.11	0.302	0.825	0.18	0.297	0.826
**Neurological disorder**							
Epilepsy	0.35	0.28	0.465	0.894	0.39	0.457	0.884
Migraine	2.89	1.08	0.197	0.631	2.11	0.245	0.627
Multiple sclerosis	0.02	0.02	0.644	0.970	0.03	0.567	0.957
Parkinson's disease	0.11	0.11	0.567	0.943	0.14	0.537	0.936
Dementia	0.03	0.16	0.111	0.737	0.22	0.090	0.723
**Orthopedic disorder**							
Osteoporosis	3.96	1.75	0.239	0.648	3.03	0.291	0.656
Rheumatoid arthritis	0.71	0.79	0.403	0.834	1.25	0.347	0.801
Osteoarthritis	3.83	9.98	0.249	0.614	17.42	0.204	0.631
Gout	3.86	3.22	0.462	0.815	5.38	0.499	0.838
**Urinary disorder**							
Chronic kidney disease	0.13	0.90	0.183	0.832	1.18	0.148	0.817
Renal stone	6.34	2.54	0.315	0.732	5.02	0.435	0.770

eTable 3. Tetrachoric correlation between self-reports and claims records of chronic diseases using ICD-9 and ICD-10

	ICD-9	ICD-10
**Cardiometabolic disorder**		
Valvular heart disease	0.597	0.571
Coronary artery disease	0.789	0.845
Arrhythmia	0.632	0.652
Cardiomyopathy	0.576	0.662
Congenital heart disease	0.472	0.595
Hyperlipidemia	0.681	0.752
Hypertension	0.949	0.949
Stroke	0.803	0.838
Type II Diabetes Mellitus	0.969	0.971
**Respiratory disorder**		
Asthma	0.709	0.675
Chronic obstructive pulmonary disease	0.418	0.444
**Gastrointestinal disorder**		
Peptic ulcer disease	0.519	0.534
Gastroesophageal reflux disease	0.634	0.641
Irritable bowel syndrome	0.492	0.493
**Psychiatric disorder**		
Schizophrenia	0.962	0.946
Bipolar disorder	0.735	0.750
Major depressive disorder	0.762	0.755
Substance use disorder	0.704	0.631
Obsessive-compulsive disorder	0.837	0.821
**Neurological disorder**		
Epilepsy	0.915	0.871
Migraine	0.634	0.626
Multiple sclerosis	0.977	0.962
Parkinson's disease	0.962	0.924
Dementia	0.666	0.719
**Orthopedic disorder**		
Osteoporosis	0.915	0.871
Rheumatoid arthritis	0.831	0.836
Osteoarthritis	0.601	0.634
Gout	0.804	0.829
**Urinary disorder**		
Chronic kidney disease	0.871	0.790
Renal stone	0.721	0.734

ICD, International Classification of Diseases.

eMaterial 1. Supplemental methods, results, and discussion

Methods

Several sensitivity analyses and subgroup analyses were conducted. First, we calculated Cohen's kappa statistic, representing a chance-adjusted agreement. Based on Landis and Koch's classifications, the interpretation of kappa was ≤0.2 as slight, 0.21–0.40 as fair, 0.41–0.60 as moderate, 0.61–0.80 as good, and 0.81–1.00 as almost perfect.[6] However, since disease prevalence significantly affected the kappa value, we did not include this finding in the main text.[7]

Second, the timeframe for self-reported health conditions is not well-defined. We opted for a 2-year observational period prior to the initial interview date, acknowledging that remote diagnoses, which were not treated in recent years, might be overlooked by patients. This period was chosen somewhat arbitrarily, and to accommodate potential variability, we conducted a sensitivity analysis using a 5-year observational period.

Finally, given that the National Health Insurance (NHI) claims data included International Classification of Diseases, 9th revision (ICD-9) and 10th revision (ICD-10) codes during the study periods, we performed a subgroup analysis using different datasets: data from 2015 and earlier were using ICD-9, and data from 2018 onwards under ICD-10. To maintain consistency, 2016 and 2017, which contained a mix of ICD-9 and ICD-10 data, were excluded from this subgroup analysis.

Results and Discussion

Kappa values ranged from 0.039 to 0.739, showing good concordance for certain conditions, including hypertension, type II diabetes mellitus, and multiple sclerosis (see eTable 2). Conversely, the concordance for valvular heart disease, cardiomyopathy, and congenital heart disease was only slight, as indicated by the low kappa statistics. This lower agreement could be attributed to the relatively low prevalence of these specific diseases.[7] Furthermore, study participants may have occasionally misidentified normal variations (eg, mild mitral valve prolapse or sinus arrhythmia) as pathological conditions, leading to a higher percentage of self-reported cases than those recorded in the claims data.

Using 5-year observational periods in the NHI claims database resulted in a higher prevalence of recorded diagnoses compared to 2-year periods. The kappa and tetrachoric correlations for the 5-year period may slightly improve or worsen compared to those for the 2-year period. However, conditions with high concordance in the 2-year period analysis remained high in the 5-year period, while those with low concordance persisted across both 2- and 5-year observational periods (See eTable 2).

eTable 3 presents the subgroup analysis of the tetrachoric correlations between self-reports and claims records using ICD-9 and ICD-10, revealing generally consistent results. However, an exception was noted for congenital heart diseases, with a correlation of 0.595 under ICD-10 compared to 0.472 under ICD-9. This might be due to ICD-10 providing more comprehensive codes that align more closely with patients' self-reports.
